# Disseminated hydatid cyst disease; a rare presentation in a tertiary care hospital of Azad Jammu Kashmir

**DOI:** 10.1016/j.ijscr.2022.107578

**Published:** 2022-08-31

**Authors:** Sarosh Khan Jadoon, Raja Mohammad Ijaz Khan, Aisha Khan Jadoon, Arti, Rahul Robaish Kumar, Abdul Majeed, Rahul Kumar, Sahil, Manjeet Singh, Muhammad Sohaib Asghar

**Affiliations:** a General Surgery Department, SKBZ CMH Muzaffarabad, Azad Jammu, And Kashmir, Pakistan; b General Surgery, SKBZ CMH Muzaffarabad, Azad Jammu, And Kashmir, Pakistan; c Ob/Gynae Department, Aga Khan University Hospital, Karachi, Pakistan; d Jinnah Sindh Medical University, Karachi, Pakistan; e Liaquat College of Medicine and Dentistry, Karachi, Pakistan; f Ghulam Muhammad Mahar Medical College Sukkur, Pakistan; g Chandka Medical College, Larkana, Pakistan; h Liaquat National Hospital and Medical College, Karachi, Pakistan; i Department of Internal Medicine, Dow University of Health Sciences-Ojha Campus, Karachi, Pakistan

**Keywords:** Echinococcosis, Hydatid cyst, Case report

## Abstract

Cystic Echinococcosis (CE) is one of the many neglected animal-based diseases also called zoonotic, it has been highlighted by the World Health Organization. It is a human and animal health issue in numerous endemic regions around the globe. CE is caused by the tapeworm Echinococcus granulosus.

We will discuss a case of a 36 years old female from a rural area who presented to the emergency department with abdominal distension, fever, and shortness of breath. History revealed that she reared sheep in her village so ELISA for anti-Echinococcus antibodies shows positive titers, and CT scan revealed type IIA and III hydatid cysts in the lungs, chest, liver, spleen, abdomen, and pelvis. An extensive exploratory laparotomy was performed and an effort was made to remove all cysts from the liver, pelvis, spleen, and abdomen. The patient remained in a vegetative state post-operatively on ventilator support. She passed away five days post-surgery. As per author's knowledge, there has not been a similar case reported as yet.

## Highlights

• We present a case of middle aged female with multiple large calcified hydatid cysts throughout thoracic and abdominal cavities.
• This has also been significantly underreported.
• As per author’s knowledge there has not been a similar case reported.

## 1 Introduction

CE is a larval stage disease of a small tapeworm (Echinococcus granulosus) that may result in infection in animals and humans. It is endemic in Asia and causes fatal health issues. The annual infection rate worldwide is 1.2 million, with a mortality of 2.2 % per annum. Risk factors for human infection include poor hygiene, contaminated food, water, and consumption of raw vegetables. Domestic or wild animals are definitive hosts, whereas livestock and humans act as intermediate hosts. Slaughter of livestock in open spaces where dogs and other cattle drop feces and also feed on infected offal [1]. We hereby present an interesting case report of a middle-aged female with multiple large calcified hydatid cysts throughout thoracic and abdominal cavities.

## 2 Case report

A 36-year-old female resident of Azad Kashmir belonging to a lower socioeconomic status with no known co-morbid, presented to the emergency department with the complaint of abdominal distension/protrusion for five years, abdominal pain for twenty days, and undocumented fever along with shortness of breath and non-productive cough for a week. She also had non-projectile vomiting for 2 days. Her symptoms were not exaggerated with food intake neither did she have any other associated issues. In medical history, it was found that she had lived up to abdominal distension for five years which led to gradual deterioration of her functionality. She was diagnosed to have worm infestation a couple of years back for which she sought medical treatment. Her bowel was regular but had burning micturition, increased frequency, and nocturia for a week in addition to incontinence on and off, she had a loss of appetite recently without significant weight drop over the past couple of months. She had no addiction or allergies although she reared sheep in her village. Gynecological/obstetrical revealed that she had secondary amenorrhea for 5 years. She is Para 4 + 0. Her last delivery was spontaneous vaginal 6 years ago. She did not have any significant surgical, blood transfusion, or family history.

She was vitally stable at the time of admission. On examination was conscious and well oriented in a time person place. Vitals: BP 120/70 mm/hg, Pulse 76/min, Temperature afebrile, Oxygen saturation 96 % at room air, Respiratory rate 20/min. Anemia++, jaundice-, koilonychia-, edema-, dehydration-, lymph nodes-, JVP raised, Breast = soft non-tender, Central nervous system = Glasgow coma scale 15/15, no fits or headache, Cerebrovascular system = S1 + S2 audible, Respiratory system = normal vesicular breathing, Bilateral decreased air entry, basal crept, Abdomen = distended, tense, tender, distended with multiple palpable variable size nodules. Borborygmi +, Reflexes normal. Antibiotics, painkillers, albendazole 400 mg (two times a day), and heparin started. Ultrasound abdomen was done which showed multiple cysts with daughter cysts involving multiple viscera, hepatosplenomegaly, and bilateral hydronephrosis.

**Fig. 1:**
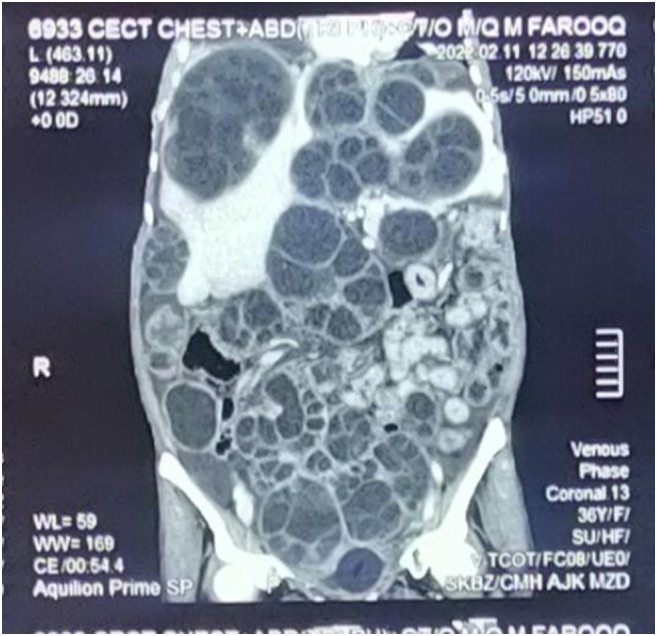
Longitudinal sections of CT scan.

**Fig. 2:**
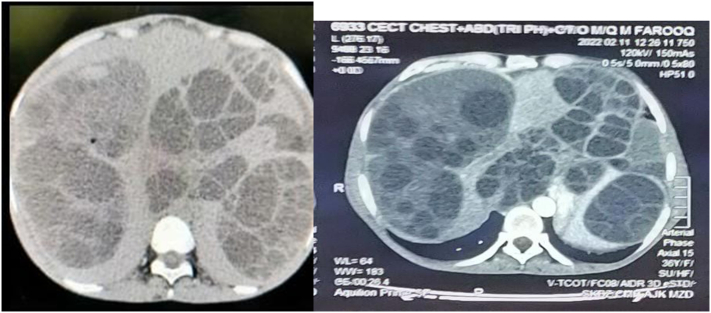
Transverse section of CT scan.

Investigations on the day of admission: Hemoglobin 8.7 g/dL, Total leukocyte count 10.5 × 10^9^/L, with 85 % neutrophils, platelet count 340 × 10^9^/L while rest of the laboratory investigations including liver function tests, renal function test, serum electrolytes, urine analysis, prothrombin time/Activated partial thromboplastin time were normal. Urea 10.6 mg/dL, creatinine 1.26 mg/dL, Hepatitis B and C serology non-reactive, serum albumin 2.9 g/L, Echinococcus IgG ELISA (enzyme-linked immunosorbent assay) titers positive. Echocardiography showed an ejection fraction of 50–55 % with pleural effusion and right-sided chamber collapse. CT scan brain was unremarkable. CT Chest showed calcified daughter cyst measuring 5.7 × 4.8 × 3.2 cm, no pleural effusion, and multiple media basal segment daughter hydatid cysts (Fig. 1). Abdomen had type IIA and III cysts displacing gut loops, splaying vessels and compressing viscera, and gall bladder distended as shown in Fig. 2. Kidneys showed bilateral moderate hydronephrosis and mild ascites. Urinary bladder and pancreas were normal.

**Fig. 3:**
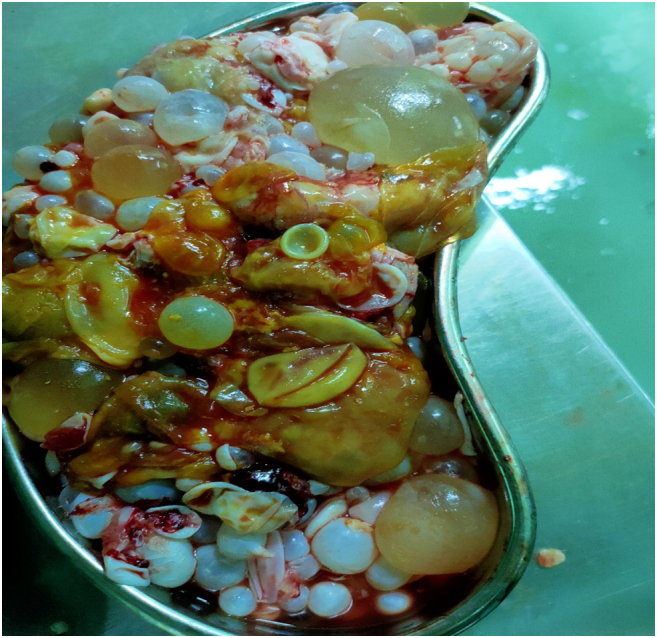
Multiple cysts extracted.

**Fig. 4:**
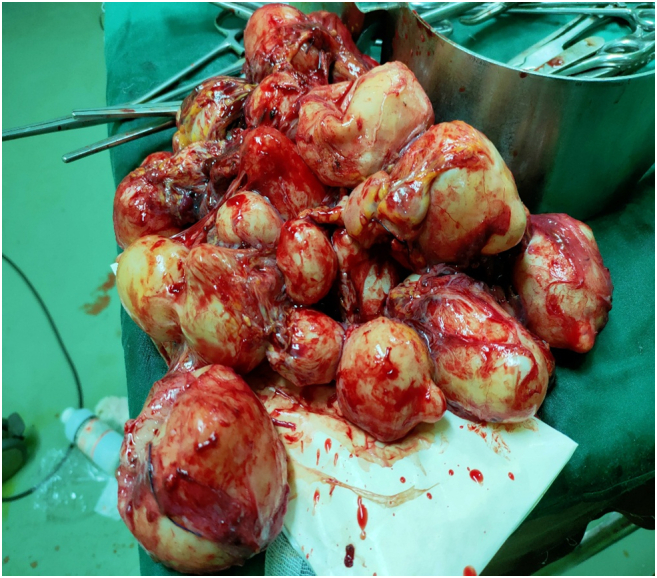
Calcified cysts.

She was shifted under surgical department care on 12th day of admission. She was vitally stable maintaining oxygen saturation at 94 % at room air. Hemoglobin 12.6 g/dL, total leukocyte count 12.9 × 10^9^/L, platelet count 465 × 10^9^/L after multiple iron infusions and one pint packed cell volume transfusion, thyroid stimulating hormone was normal. Digital rectal examination was performed that was unremarkable. On per vaginal examination, vulva/vagina normal appearing with no signs of prolapse, fullness in pelvic area, cervical os closed, no vaginal discharge or bleeding. An exploratory laparotomy was performed. Operative findings were multiple small and large-sized cysts involving mesenteric border of bowel (Fig. 3), and therefore, pericystectomy was done. Multiple cysts in the left lobe and right lobes of liver, pelvis, and spleen, as shown in Fig. 4 (all were removed). Perioperatively 5 pint packed cell volumes, 4 fresh frozen plasma, 1 whole blood was transfused, and she was put on ventilator inotropic support with 2 drains. Tazobactum and metronidazole (antibitotics) were started. She remained hemodynamically unstable on and off till her demise after 5 days of surgery due to sepsis.

## 3 Discussion

Pakistan is a third-world country lying under low socioeconomic status. It is extremely populated where the majority reside in rural areas or jam-packed urban areas with awful sanitary facilities. A big number earn their living through agriculture and local dairy farming on a minute scale; where they come into close contact with animals and thus, putting themselves unknowingly at the risk of acquiring echinococcus infection. This can be easily avoided by proper health and hygiene measures.

Adult tapeworms live inside the small intestine of definitive hosts, hydatid cyst stages occur in herbivorous intermediate hosts. Tapeworm eggs are defecated by an infected dog and ingested by cattle; which hatch into embryos inside intestine, penetrate intestinal lining, and later through the bloodstream to organs where they transform and develop into larval echinococcal cysts in which numerous tiny tapeworm heads called protoscolices are produced via asexual reproduction. One cyst can have thousands of protoscolices, each protoscolex can develop into an adult tapeworm when ingested by the definitive host [2].

Hydatid cysts can affect any part of the body and can be found as single or multiple in numbers. Many times, hydatid cysts are found as the incidental finding and remain asymptomatic [3]. Literature search embarked on us that echinococcus is most common in liver (70 %, 25 %) and lungs (25 %, 4 %) [3], [4]. Involvement of the neck is a much rare condition involving 0.75 % of all cases [5]. In our case it was found to be in lungs, liver, pelvis as well as neck. In vascularized bone in up to 4 % of cases, the kidneys (3 %), thyroid gland (2 %), ovary 0.2–1 % [6], heart 0.5–2 % [7]. Prevalence of multiple pulmonary cysts and bilateral cysts is 30 % and 4 % [3]. We found no data to date from Pakistan reporting a case of hydatid cyst in a neck. Research shows that lung cysts grow much faster than others because of negative pleural pressure. A cough, dyspnoea, and hemoptysis can be the typical features of hydatid cyst affecting the lung, and hepatomegaly with abdominal pain can be the presenting features of hydatid cyst affecting the liver, just as this case we presented, except for hemoptysis [3].

The case we are presenting resided in a rural area of Kashmir that reared sheep and according to research conducted in Pakistan in 2021, the hydatid cyst infection was highly prevalent in buffaloes (12 %), followed by sheep (10 %), cows (9 %), and goats (5.1 %). It was more prevalent (10 %; 96/992) in district Lakki Marwat followed by district Bannu (9 %; 112/1246) and Karak (7 %; 39/595) [8], and these regions are not very far away from Kashmir.

Diagnosis of hydatid cyst can be established with a combination of medical and family history, patient's residency, occupation, physical examination, radiological evaluation, laboratory diagnosis, serological tests, and histopathological assessment by using a CT-guided biopsy. The indirect haemagglutination test and ELISA have a sensitivity of 80 % to diagnose hydatid cysts but ELISA test is also useful to detect recurrence [3]. CT scan is used to find out the calcification of the cyst. Ultrasonography has 90–95 % sensitivity and specificity. The presence of eosinophilia is not specific. Aspiration cytology is typically contraindicated due to the risk of rupture and anaphylactic shock which is much lower by fine-needle aspiration declared as a gold standard diagnostic technique. Management depends upon the size, site, extent and inculcates medical treatment with mebendazole or albendazole 400 mg BID for 3–6 months with strict follow-up, needle aspiration under ultrasonography, minimally invasive techniques, and open surgical/ laparotomy approaches [3], [5].

Surgery is the gold standard approach with the use of pre- and post-operative antiparasitic drugs as prophylaxis to decrease the risk of contamination and recurrences. There exists a high risk of anaphylactic shock if the cyst ruptures due to pressure exerted during surgical excision or trauma to the body.3,5 Similar event happened during surgery of the patient that we operated on, besides all careful steps the trauma inflicted on the body could not be avoided. This patient had put us in a do or die situation. Timely management through gold standard technique was laparotomy but due to chronic extensive diffused hydatid cyst disease the mortality could not be avoided. A similar case was reported by Bairwa et al. in a 12 year old boy but they opted for laparosocopic approach with good results [9].

We reported this care report according to the guidelines provided by SCARE group, while consent was provided by the family to report the findings [10].

## 4 Conclusion

Surgical management of hepatic hydatid cysts is usually safe and effective. Laparoscopic approach with optimum technique of controlled suctioning over cyst content is recommended. However, surgery combined with oral antihelminthics is the mainstay of treatment for hydatid cysts. But with cases involving extensive multivisceral cysts, as present in our case, surgical management can be challenging with high post-operative mortality.

## Provenance and peer review

Externally peer reviewed, not commissioned.

## Consent statement

Written informed consent was obtained from the patient's family for publication of this case report and accompanying images. A copy of the written consent is available for review by the Editor-in-Chief of this journal on request.

## Funding

None.

## Ethical approval

Not applicable.

## Registration of research studies

Not applicable.

## Guarantor

MUHAMMAD SOHAIB ASGHAR

## CRediT authorship contribution statement

Writing the paper & assisting in the procedure: PROF. DR. RAJA MOHAMMAD IJAZ KHAN, SAROSH KHAN JADOON.

Performing the procedure & assisted in Literature search: AISHA JADOON.

Review of the manuscript: MUHAMMAD SOHAIB ASGHAR, MANJEET SINGH.

Assisted in writing manuscript: ARTI, RAHUL ROBAISH KUMAR,

Designed the study: ABDUL MAJEED, RAHUL KUMAR.

Literature Review: SAHIL, MUHAMMAD SOHAIB ASGHAR.

Guarantor: MUHAMMAD SOHAIB ASGHAR.

## Declaration of competing interest

None.
